# Interesting read

**DOI:** 10.3205/zma001481

**Published:** 2021-04-15

**Authors:** Sören Huwendiek

**Affiliations:** 1 University of Bern, Medical Faculty, Institute for Medical Education, Department for Assessment and Evaluation, Bern, Switzerland

## Editorial

Although this issue of the GMS J for Med Educ does not follow a fixed main theme, the contributions of the current issue can be assigned to four topics: communication; digital skills & online teaching; simulation; ethical or scientific thinking.

Three of the papers deal with the topic of communication. Two of these are dedicated to the teaching of these competences: one in the field of human medicine on the basis of a teaching concept for conversation [1] and one from the field of veterinary medicine, which also represents a position paper of our professional society (Society for Medical Education, GMA) [2]. The third contribution also examines the topic of assessing communication more closely on the basis of standardised patients [3]. Here, standardised patients who were dialogue partners and external standardised patients assessed the students, the latter via video recording. Personally, I find the assessment of the patient's perspective to be important, especially with respect to the assessment of doctor-patient communication. 

Furthermore, there are three contributions on the topics of digital competences and online teaching. The work of Aulenkamp et al. [4] deals with a Germany-wide survey of teaching events on the theme of “digital competences”. This refers to knowledge in dealing with data sets, telemedicine or apps, but not digitalised teaching formats, which are addressed by the other two articles in this topic area. The results of this survey show a clear need for further expansion of such teaching events. The article by Langewitz et al. [5] deals with an online doctor-patient communication training course conducted with trained standardised patients, in which the standardised patients provide much appreciated feedback to the students. In the article by Streitlein-Böhme et al. [6], experiences with the digitalisation of a practical year seminar in general medicine are presented.

Four contributions can be assigned to the topic of “simulation”. The article by Guinez-Molinos et al. [7] evaluates the feasibility and acceptance of assessment tools to measure interpersonal, collaborative and clinical competences in cardiac emergency scenarios for medical students. The article by Lottspeich et al. [8] explains how the central competence of ward rounds can be taught in a structured way and presents guidelines for establishing such a course. The article by Łoś et al. [9] examines the extent to which technical and non-technical skills of medical students in a paediatric emergency course with high-fidelity simulations are related to mindfulness and stress. The article by Kasselmann et al. [10] presents the (sobering) results of a nationwide survey on the implementation of disaster medicine offerings in medical school. 

Two articles in this issue deal with ethical and scientific thinking, respectively. The article by Kuhn et al. [11] deals with the establishment of an extracurricular offer for medical students from the 5th clinical semester onwards, as well as in the practical year and for residents, in order to support the handling of ethical dilemmas in everyday clinical life, among other things through principle-oriented case conferences. In my opinion, such exemplary courses should be offered firmly anchored in the curriculum in the future. The article by Schmidt et al. [12] examines the extent to which scientific thinking and statistical skills are present in practising physicians and how these skills were acquired. Not unexpectedly, the physicians’ own research experience seems to be relevant for the acquisition of these skills.

I think the 12 papers in this issue represent a nice excerpt of central topics in our field. 

I hope you enjoy this interesting read!

## Competing interests

The author declares that he has no competing interests.
